# Drivers of socioeconomic inequalities of child hunger during COVID-19 in South Africa: evidence from NIDS-CRAM Waves 1–5

**DOI:** 10.1186/s12889-022-14482-1

**Published:** 2022-11-16

**Authors:** Olufunke A. Alaba, Charles Hongoro, Aquina Thulare, Akim Tafadzwa Lukwa

**Affiliations:** 1 Health Economics Unit, School of Public Health and Family Medicine, Faculty of Health Sciences, University of Cape Town, Anzio Road, Observatory, 7925 South Africa; 2 Developmental, Capable and Ethical State, Human Sciences Research Council, Pretoria, Gauteng Province South Africa; 3 School of Health Systems and Public Health (SHSPH), Faculty of Health Sciences, University of Pretoria, Pretoria, Gauteng Province South Africa; 4 National Department of Health, Pretoria, Gauteng Province South Africa; 5 DSI-NRF Centre of Excellence in Epidemiological Modelling and Analysis (SACEMA), Stellenbosch University, Private Bag X1, Matieland, Stellenbosch, 7602 South Africa

**Keywords:** Child hunger, Food (in)security, Covid-19

## Abstract

**Background:**

Child hunger has long-term and short-term consequences, as starving children are at risk of many forms of malnutrition, including wasting, stunting, obesity and micronutrient deficiencies. The purpose of this paper is to show that the child hunger and socio-economic inequality in South Africa increased during her COVID-19 pandemic due to various lockdown regulations that have affected the economic status of the population.

**Methods:**

This paper uses the National Income Dynamics Study-Coronavirus Rapid Mobile Survey (NIDS-CRAM WAVES 1–5) collected in South Africa during the intense COVID-19 pandemic of 2020 to assess the socioeconomic impacts of child hunger rated inequalities. First, child hunger was determined by a composite index calculated by the authors. Descriptive statistics were then shown for the investigated variables in a multiple logistic regression model to identify significant risk factors of child hunger. Additionally, the decomposable Erreygers' concentration index was used to measure socioeconomic inequalities on child hunger in South Africa during the Covid-19 pandemic.

**Results:**

The overall burden of child hunger rates varied among the five waves (1–5). With proportions of adult respondents indicated that a child had gone hungry in the past 7 days: wave 1 (19.00%), wave 2 (13.76%), wave 3 (18.60%), wave 4 (15, 68%), wave 5 (15.30%). Child hunger burden was highest in the first wave and lowest in the second wave. The hunger burden was highest among children living in urban areas than among children living in rural areas. Access to electricity, access to water, respondent education, respondent gender, household size, and respondent age were significant determinants of adult reported child hunger. All the concentrated indices of the adult reported child hunger across households were negative in waves 1–5, suggesting that children from poor households were hungry. The intensity of the pro-poor inequalities also increased during the study period. To better understand what drove socioeconomic inequalites, in this study we analyzed the decomposed Erreygers Normalized Concentration Indices (ENCI). Across all five waves, results showed that race, socioeconomic status and type of housing were important factors in determining the burden of hunger among children in South Africa.

**Conclusion:**

This study described the burden of adult reported child hunger and associated socioeconomic inequalities during the Covid-19 pandemic. The increasing prevalence of adult reported child hunger, especially among urban children, and the observed poverty inequality necessitate multisectoral pandemic shock interventions now and in the future, especially for urban households.

## Background

Hidden hunger afflicts several people around the world [1] and has a number of adverse effects, including poor health, stunted growth, reduced productivity, intellectual disability and unexpected death. Child hunger undermines a child's health and survival, and can have severe effects, particularly from conception to her second year of life or within the first 1,000 days of life, characterised by severe cognitive and physical consequences [2]. Every child deserves a healthy start in life. But in Africa, too many starving children, where hunger is the norm, suffer from chronic pain [3]. Looking at the prevalence of food insecurity and hunger, all aspects of child malnutrition are found in all regions of Africa [3]. Child malnutrition is prevalent in sub-Saharan Africa, including Ethiopia, South Sudan, Somalia, Kenya, Burundi, Niger, Zambia, East Africa, West Africa, and Southern Africa [3–5]. The emergence of diseases such as Ebola virus, Zika virus, Chikungunya virus and the coronavirus COVID-19 has been argued to have exacerbated the hunger crisis [6–11].

Many stakeholders around the world highlighted the threat of an imminent global food crisis [12] due to the impact of the novel coronavirus (COVID-19). Africans were exposed to more severe adverse effects emanating from their food systems due to the COVID-19 pandemic [12]. In major African cities (Nairobi, Kinshasa and Lagos), about two-thirds of the population depends on the informal sector for their livelihoods [12]. However, the shock of the COVID-19 pandemic drove up unemployment, exacerbated poverty, and ultimately had a direct impact on hunger and ill health [13]. Child hunger has a consistent and significant impact on economic growth.

Many countries, including South Africa, enacted strict lockdown measures which prohibited movement. As a result, jobs were lost, unemployment rose, and disposable income fell. As a result, many lived below the poverty line and could not afford basic food items [14]. In addition, the secondary economy, which consisted mainly of informal jobs that provide a daily income, were hit hard. Restrictions disrupted agricultural supply chains, resulting in higher food prices that households could not afford, a situation similar to the 2014 Ebola crisis in West Africa [12]. In addition, health protection measures such as quarantine and social distancing enforced during the COVID-19 pandemic forced poor people to access their farms and off-land food systems which were impossible to access. As highlighted in the reviewed literature, the emergence of highly contagious and deadly infectious diseases in times of drought and famine completely undermines the vulnerable food security of the poor [15–17].

The COVID-19 pandemic had a major impact on food, nutrition and health security of vulnerable groups (young children, pregnant and lactating women) and exacerbated social and health inequalities [18, 19]. According to the World Food Programme, draconian lockdown measures to curb the spread of COVID-19 such as closing of schools, resulted in more than 368 million schoolchildren missing school lunches [11, 20]. About 50% of these children resided in low- and middle-income countries. Loss of access to school meals threatened children's health, impacted household food security, and impacted the most vulnerable households whose disposable income had decreased due to the COVID-19 pandemic [13]. Childhood hunger has long-term and short-term consequences, and children who suffer from hunger are known to be at risk of many forms of malnutrition, including wasting, stunting, obesity, and micronutrient deficiencies [21–24]. South Africa has suffered high levels of hunger in the past [7].

Before the COVID-19 outbreak in South Africa, around 16% of households had reported inadequate access to food and 5.5% of the population had reported very inadequate access to food. About 11% of households had reported being vulnerable to hunger [25]. Reported hunger is one of the key indicators for monitoring food insecurity at the household level. In 2018, her 11% (2.1 million) of South African children lived in households that reported child hunger [26]. Self-reported child hunger in South Africa has been reported to have decreased significantly, but stunting (an indicator of chronic malnutrition) remains very high for an upper middle-income country [5].

Household hunger increased dramatically under stringent lockdowns due to COVID-19, with 47% of households running out of money to buy groceries in May/June 2020 (first wave), while child and adult hunger declined by 15% and increased by 22% [27]. The proportion of households experiencing hunger dropped to 37%. However, hunger and food insecurity remained significantly higher than before COVID-19 [6]. Furthermore, it has been argued that the COVID-19 pandemic could have pushed about 49 million people into extreme poverty by the end of 2020 [10]. Socioeconomic status is a major determinant of hunger, so the more people who suffer from hunger, the less likely it is to reach zero hunger by 2030 [28]. It has been argued that this unequal distribution of all forms of hunger and malnutrition is rooted in inequalities of social, political and economic power [29]. Therefore, the first step in addressing hunger inequalities is to understand how hunger inequalities are embedded and reinforced in power inequalities in the food system. The purpose of this paper was to examine whether the burden of child hunger and socioeconomic inequality in South Africa increased during the COVID-19 pandemic.

## Methods

### Data

During the COVID-19 crisis, South Africa joined the international community in the fight against COVID-19 by adopting strict lockdown measures. Large-scale personal data collection activities were ceased during the covid-19 pandemic. NIDS-CRAM is a rapid telephone survey derived from the nationally representative existing household panel survey, the National Income Survey (NIDS^1^). The NIDS-CRAM survey sampled individuals from NIDS wave 5. Unlike previous waves of NIDS, NIDS-CRAM did not attempt to interview or collect information on everyone currently living with the sampled individual [25]. However, the change in the sampling protocol was carefully considered taking into account the main goals and constraints of the NIDS-CRAM [25]. Wave 1 was captured during lockdown phases 3 and 4 of the nationwide lockdown, wave 2 was captured during the 'advanced' phase 3 of the lockdown, and finally wave 3 was captured during phases 2 and 1. Based on responses to adult reported child hunger questions (children under 18), the final sample considered in the study was 5652, 4476, 4429, 4,208 and 4,341 individuals for waves 1–5, respectively [30–32]. This study only considered individuals who responded to the question about childhood hunger in the past 7 days in the analysis. Each wave occurred at a specific time and duration with; Wave 1 between (May–June 2020), Wave 2 between (July–August 2020), Wave 3 between (November and December 2020), Wave 4 between (February–March 2021), and Wave 5 between (April–May 2021). Wave 1 was collected during stages 3 and 4 of the national lockdown, wave 2 was collected during the 'advanced' stage 3 of the lockdown, wave 3 was collected during stage 2, and wave 4 was collected during stages 2 and 1 and finally wave 5 was collected in Stage 1 [33].

### Outcome variable: child hunger

In all five waves, respondents were asked a question "In the past seven days, has any child under 18 years in your household gone hungry because there was not enough food?" and "In last seven days, has anyone in your household gone hungry due to lack of food?" [34–38]. This study then used the questions above to make a composite index, which was used as a proxy to estimate the adult reported child hunger among children. Both questions had binary responses "yes" or "no", see Table 1 on how the composite index was made. See Table 2 which shows the determinats included in the regression analysis.

**Table 1:** Computation of the Child Hunger index among children

Child Hunger (yes) coded as 1	If a child had gone hungry in the last 7 days (yes) coded as 1 and residing in the household that had gone hungry in the last 7 days (yes) coded as 1
	If a child had gone hungry in the last 7 days (yes) coded as 1 and residing in the household that had not gone hungry in the last 7 days (no) coded as 0
Child of Hunger (no) coded as 0	If a child had not gone hungry in the last 7 days (no) coded as 0 and a child residing in the household that had gone hungry in the last 7 days (yes) coded as 1
	If a child had not gone hungry in the last 7 days (no) coded as 0 and a child residing in the household that had not gone hungry in the last 7 days (no) coded as 0

**Table 2:** Description of variables

*Dependent variable*	
Adult Reported Child Hunger (ARCH)	ARCH was dichotomized; 1 (yes), if child experienced child hunger and 0 (no) if child experienced, did not experience a child hunger
***Independent variables***	
Employment status	Employment_Status was recoded as; 0 "not economically active", 1 "unemployed" & 2 "employed"
Residence status	The residence was recorded as; 0 "rural" and 1 "urban"
Race	The race was retained as it was; 1 "African/Black", 2 "Coloured", 3 "Asian/Indian" and 4 "White"
Socio-economic Status (SES)	SES was household income which was grouped into 5 categories and coded as; 0 "poorest", 1 "poorer", 2 "middle", 3 "richer" & 4 "richest"
Dwelling Type	Dwellintype was coded as; 0 "House/Flat", 1 "Traditional/Mud" and 2 "Informal/Shack"
Electricity access	ElectricityAccess was coded as 1 "yes" and 2 "no"
Piped-water access	PipedWaterAccess was coded as 1 "yes" and 2 "no"
Respondent's Education Level	RespondentEdu was recoded into 5 categories; 0 "no schooling", 1 "primary", 2 "secondary", 3 "tertiary"
Gender	Gender was retained. It was as 1 "male" and 2 "female"
Household size	HseHoldSize was retained as it was "a continuous variable"
Respondent's age	Respondent Age was retained as it was "a continuous variable"

### Analytical methods

#### Analysis of the association of the predictors with the outcome variables

To predict the dependent variable, adult reported child hunger burden, we used binary logistic regression across five waves. Binary logistic regression is most useful when the dependent variable is dichotomous [39]. This study used logistic regression to calculate odds ratios for all independent variables for each independent variable category, excluding the reference category, to assess associations between adult reported child hunger burden and demographic variables. We used the Erreygers-normalized concentration index to estimate health inequalities in terms of adult reported child hunger and the causes of health inequalities among children in South Africa across five waves. The logistic regression results were used to construct and decompose the normalized Erreygers concentration index. The Erreygers Normalized Concentration Index is described under the Concentration Curves subheading.

### Concentration curves and indices

The Concentrated Index approach is a standard measure for assessing income-related health inequalities. Concentration indices and curves are commonly used to identify socioeconomic inequalities in health variables. In this article, we used the Erreygers [40] normalized concentration index to measure the degree of socioeconomic inequality in hunger and undernutrition among children in South Africa during the COVID-19 pandemic. Of the many indices that could have been used, we chose to adopt the Ellergar as it is the most likely modified version of the index and therefore provides more robust results.

The concentration index can be computed by making use of the 'convenient covariance' as shown below:

1 $$CI=\frac{2}{\widehat{y}} COV \left({y}_{i},{R}_{i}\right)\cdots \cdots \cdots \cdots \cdots \cdots \cdots$$

where: y_i_ is the health variable.

ŷ is the mean of y_i_.

R_i_ is the fractional rank of the ith individual.

COV denotes the covariance.

The concentration index is calculated as twice the area between the concentration curve and the isometric line (45 degree line) [41]. The absence of inequalities in health is reflected in the concentration curve lying on the 45° line. The degree of health disparities is indicated by how far the concentration curves deviate from the isoline (45° line). The further the concentration curve is from the the isoline, the greater the magnitude of health inequalities [42]. Therefore, a true zero for the normalized Erreygers concentration index indicates no socioeconomic inequality.

In contrast, negative values ​​indicate a disproportionate concentration of socioeconomic inequality among the poor, while positive values ​​reflect a concentration of socioeconomic inequality among the rich [43, 44]. Erreygers [40, 45] argued that normalizing the formula for the health concentration index reliably solves the boundary problem for binary health variables, so we decided to use the normalized formula in this study. The Erreygers normalized index (E(c)) can be expressed as:

2 $${E}_{c} = \frac{4\widehat{y}}{{y}^{max} - {y}^{min}} CI\cdots \cdots \cdots \cdots \cdots \cdots \cdots$$

where y^max^—y^min^ is the range of the health variable, which is 'one' in the case of binary variables. The study used reported total household income after tax in the concentration indices and curve computations. The Erreygers normalized concentration index was later decomposed to understand better what was driving the inequalities.

## Results

### Demographic characteristics

The overall burden of child hunger rates varied across the five waves (1–5). With proportions of adult respondents indicated that a child had gone hungry in the past 7 days: wave 1 (19.00%), wave 2 (13.76%), wave 3 (18.60%), wave 4 (15, 68%), wave 5 (15.30%) (Table 3). The first wave was the highest and the second wave was the lowest. It was also higher for children living in urban areas than for children living in rural areas. In addition, it was highest in children from the poorest families in waves 3–5, lowest in children from richest families, and highest in children from poorer families in waves 1 and 2 (Table 3).

**Table 3:** Demographic characteristics

	**Prevalence of adult reported Child Hunger**									
Socio-demographic Characteristics	**Wave 1** *N* = *5,619 (19.00)*		**Wave 2** *N* = *4,586 (13.76)*		**Wave 3** *N* = *4,419 (18.60)*		**Wave 4** *N* = *4,030 (15.68)*		**Wave 5** *N* = *4,118 (15.30)*	
	**N (%)**	**Pearson Chi**^**2**^	**N (%)**	**Pearson Chi**^**2**^	**N (%)**	**Pearson Chi**^**2**^	**N (%)**	**Pearson Chi**^**2**^	**N (%)**	**Pearson Chi**^**2**^
***Employment status***										
Not Economically Active	242 (23.34)	0.00	159 (25.77)	0.00	195 (22.73)	0.00	152 (23.64)	0.00	173 (26.02)	0.00
Unemployed	481 (46.38)		279 (45.22)		324 (37.76)		278 (43.23)		263 (39.55)	
Employed	314 (30.28)		179 (29.01)		339 (39.51)		213 (33.13)		229 (34.44)	
***Residence status***										
Urban	765 (71.70)	0.00	383 (62.89)	0.04	533 (64.84)	0.88	402 (63.61)	0.04	396 (62.86)	0.04
Rural	302 (28.30)		226 (37.11)		289 (35.16)		230 (36.39)		234 (37.14)	
***Race***										
African/Black	1,008 (94.38)	0.00	606 (96.04)	0.00	833 (96.52)	0.00	630 (95.45)	0.00	652 (97.17)	0.00
Coloured	53 (4.96)		24 (3.80)		27 (3.13)		29 (4.39)		18 (2.68)	
Asian/Indian	2 (0.19)		1 (0.16)		2 (0.23)		1 (0.15)		1 (0.15)	
White	5 (0.47)		0 (0)		1 (0.12)		0 (0.00)		0 (0.00)	
***Socioeconomic Status (SES)***										
Poorest	185 (26.28)	0.00	97 (23.83)	0.00	59 (34.91)	0.00	172 (33.08)	0.00	184 (35.52)	0.00
Poorer	227 (32.24)		122 (29.98)		49 (28.99)		127 (24.42)		144 (27.80)	
Middle	123 (17.47)		86 (21.13)		33 (19.53)		114 (21.92)		106 (20.46)	
Richer	111 (15.77)		72 (17.69)		22 (13.02)		82 (15.77)		63 (12.16)	
Richest	58 (8.24)		30 (7.37)		6 (3.55)		25 (4.81)		21 (4.05)	
***Dwelling Type***										
House/Flat	(69.73)	0.00	430 (69.24)	0.00	593 (71.10)	0.00	428 (67.30)	0.00	429 (66.41)	0.00
Tradition/Mud	(18.54)		137 (22.06)		164 (19.66)		126 (19.81)		142 (21.98)	
Informal-Shack	(11.73)		54 (8.70)		77 (9.23)		82 (12.89)		75 (11.61)	
***Electricity access***										
Yes	1,002 (93.91)	0.13	591 (93.66)	0.16	806 (93.72)	0.10	610 (92.99)	0.07	622 (93.53)	0.06
No	65 (6.09)		40 (6.34)		54 (6.28)		46 (7.01)		43 (6.47)	
***Piped-water access***										
Yes	701 (65.64)	0.00	391 (61.97)	0.00	521 (60.72)	0.00	395 (60.21)	0.00	399 (60.00)	0.00
No	367 (34.36)		240 (38.03)		337 (39.28)		261 (39.79)		266 (40.00)	
***Respondent's Education Level***										
No schooling	41 (3.87)	0.00	28 (4.44)	0.00	37 (4.30)	0.00	41 (6.27)	0.00	42 (6.30)	0.00
Primary Education	353 (33.30)		206 (32.70)		277 (32.21)		193 (29.51)		194 (29.09)	
Secondary Education	658 (62.08)		393 (62.38)		536 (62.33)		415 (63.46)		420 (62.97)	
Tertiary Education	8(0.75)		3 (0.48)		10 (1.16)		5 (0.76)		11 (1.65)	
***Respondent’s Gender***										
Male	355 (33.24)	0.02	215 (34.07)	0.24	269 (31.17)	0.05	182 (27.58)	0.00	170 (25.34)	0.00
Female	713 (66.76)		416 (65.93)		594 (68.83)		478 (72.42)		501 (74.66)	

### Logistic regression results

All the independent variables included in the model were significant determinants of the adult reported child hunger among children in South Africa (Table 4). In waves 1 and 2, children who had unemployed [1.49(95% CI:1.49–1.50)] and employed parents [1.18(95% CI:1.17–1.19)] were more likely to experience child hunger compared to those whose parents who were not economically active. While in waves 3 [1.10(95% CI: 1.27–1.28)] and 4 [1.27(95% CI: 1.27–1.28)], children who had unemployed parents were more likely to experience child hunger compared to children who had parents who were not economically active. Additionally, children who had employed parents were less likely to experience a child hunger in waves 3 [0.84(95% CI: 0.98–0.99)] and 4 [0.99(95% CI: 0.98–0.99)]. However, for wave 5, children with unemployed [0.70(95% CI: 0.70 0.70)] and employed [0.74(95% CI 0.73 0.74)] parents were likely to experience a child hunger compared to children whose parents were not economically active.

**Table 4:** Logistic regression results of the adult reported child hunger

	***Wave 1***		***Wave 2***		***Wave 3***		***Wave 4***		***Wave 5***	
Socio-demographic Characteristics	Odds Ratio^1^ [Conf. Interval]^2^	Standard Error	Odds Ratio [Conf. Interval]	Standard Error	Odds Ratio [Conf. Interval]	Standard Error	Odds Ratio [Conf. Interval]	Standard Error	Odds Ratio [Conf.Interval]	Standard Error
***Employment status***										
Not Economically Active	ref	ref	ref	ref	ref	ref	ref	ref	ref	ref
Unemployed	1.49^a^ [1.49 1.50]	0.003	1.74^a^ [1.73 1.74]	0.004	1.10^a^ [1.27 1.28]	0.001	1.27^a^ [1.27 1.28]	0.003	0.70^a^ [0.70 0.70]	0.002
Employed	1.18^a^ [1.17 1.19]	0.003	1.18^a^ [1.18 1.19]	0.003	0.84^a^ [0.98 0.99]	0.000	0.99^a^ [0.98 0.99]	0.002	0.74^a^ [0.73 0.74]	0.002
***Residence status***										
Rural	ref	ref	ref	ref	ref	ref	ref	ref	ref	ref
Urban	0.98^a^ [0.98 0.99]	0.002	1.43^a^ [1.42 1.43]	0.003	1.30^a^ [1.28 1.31]	0.005	1.09^a^ [1.08 1.09]	0.002	1.28^a^ [1.28 1.29]	0.002
***Race***										
African/Black	ref	ref	ref	ref	ref	ref	ref	ref	ref	ref
Coloured	0.87^a^ [0.87 0.88]	0.002	0.75^a^ [0.74 0.75]	0.003	0.43^a^ [0.52 0.53]	0.002	0.53^a^ [0.53 0.53]	0.002	0.87^a^ [0.86 0.87]	0.003
Asian/Indian	0.28^a^ [0.28 0.29]	0.003	0.29^a^ [0.28 0.30]	0.003	0.55^a^ [0.71 0.76]	0.003	0.75^a^ [0.73 0.76]	0.008	0.20^a^ [0.20 0.21]	0.002
White	0.47^a^ [0.45 0.46]	0.002	-	-	-	-	-	-	-	-
***Socio-economic Status (SES)***										
Poorest	ref	ref	ref	ref	ref	ref	ref	ref	ref	ref
Poorer	0.54^a^ [0.53 0.54]	0.001	0.76^a^ [0.76 0.76]	0.002	1.14^a^ [1.13 1.15]	0.005	0.37^a^ [0.37 0.37]	0.001	0.68^a^ [0.67 0.68]	0.001
Middle	0.53^a^ [0.53 0.53]	0.001	0.53^a^ [0.53 0.53]	0.001	1.08^a^ [1.06 1.08]	0.004	0.51^a^ [0.51 0.51]	0.001	0.42^a^ [0.41 0.42]	0.001
Richer	0.52^a^ [0.52 0.52]	0.001	0.49^a^ [0.49 0.49]	0.001	0.51^a^ [0.50 0.51]	0.002	0.28^a^ [0.28 0.28]	0.001	0.21^a^ [0.20 0.21]	0.001
Richest	0.21^a^ [0.21 0.21]	0.001	0.20^a^ [0.20 0.20]	0.000	0.14^a^ [0.13 0.14]	0.001	0.10^a^ [0.10 0.10]	0.000	0.09^a^ [0.09 0.10]	0.000
***Dwelling Type***										
House/Flat	ref	ref	ref	ref	ref	ref	ref	ref	ref	ref
Tradition/Mud	1.05^a^ [1.04 1.05]	0.003	1.07^a^ [1.07 1.08]	0.003	1.95^a^ [1.93 1.96]	0.009	1.13^a^ [1.13 1.14]	0.003	1.66^a^ [1.65 1.66]	0.004
Informal-Shack	1.77^a^ [1.76 1.77]	0.003	0.61^a^ [0.60 0.61]	0.002	3.37^a^ [3.34 3.40]	0.014	2.17^a^ [2.16 2.18]	0.005	2.66^a^ [2.65 2.68]	0.006
***Electricity access***										
Yes	ref	ref	ref	ref	ref	ref	ref	ref	ref	ref
No	0.71^a^ [0.70 0.71]	0.002	1.96^a^ [1.95 1.98]	0.007	0.43^a^ [0.42 0.43]	0.003	0.79^a^ [0.78 0.79]	0.002	0.74^a^ [0.73 0.74]	0.002
***Piped-water access***										
Yes	ref	ref	ref	ref	ref	ref	ref	ref	ref	ref
No	1.11^a^ [1.11 1.12]	0.002	1.05^a^ [1.05 1.06]	0.002	1.13^a^ [1.12 1.14]	0.004	0.99^a^ [0.99 0.99]	0.002	0.88^a^ [0.87 0.88]	0.002
***Respondent's Education Level***										
No schooling	ref	ref	ref	ref	ref	ref	ref	ref	ref	ref
Primary Education	0.88^a^ [0.87 0.89]	0.004	0.91^a^ [0.90 0.92]	0.004	6.37^a^ [6.16 6.59]	0.107	0.85^a^ [0.84 0.85]	0.004	1.08^a^ [1.07 1.09]	0.005
Secondary Education	0.75^a^ [0.75 0.76]	0.004	0.56^a^ [0.56 0.57]	0.003	3.88^a^ [3.75 4.01]	0.065	0.70^a^ [0.70 0.71]	0.003	1.21^a^ [1.19 1.22]	0.006
Tertiary Education	0.60^a^ [0.59 0.61]	0.005	0.04^a^ [0.03 0.04]	0.001	8.91^a^ [8.53 9.30]	0.195	0.79^a^ [0.78 0.81]	0.007	2.11^a^ [2.07 2.15]	0.019
***Respondent's Gender***										
Male	ref	ref	ref	ref	ref	ref	ref	ref	ref	ref
Female	1.18^a^ [1.18 1.19]	0.002	1.02^a^ [1.02 1.02]	0.002	1.43^a^ [1.42 1.44]	0.005	0.97^a^ [0.97 0.97]	0.002	1.17^a^ [1.16 1.17]	0.002
***Household Size***	1.06^a^ [1.06 1.06]	0.000	1.04^a^ [1.03 1.04]	0.000	1.03^a^ [1.02 1.03]	0.001	1.11^a^ [1.11 1.11]	0.000	1.14^a^ [1.13 1.14]	0.000
***Respondent’s Age***	1.01^a^ [1.01 1.01]	0.000	1.01^a^ [1.01 1.01]	0.000	1.02^a^ [1.01 1.02]	0.000	1.01^a^ [1.01 1.01]	0.000	0.99^a^ [0.99 1.00]	0.000

^1^ a and b indicate statistical significance at the 1 and 5%, respectively

^2^ Confidence Interval

In all the waves; wave 2 [1.43(95% CI: 1.42–1.43)], wave 3 [1.30(95% CI: 1.28–1.31)], wave 4 [1.09(95% CI: 1.08–1.09)], wave 5 [1.28(95% CI: 1.28–1.29)] children residing in urban households were more likely to experience child hunger except for wave 1 [0.98(95% CI: 0.98–0.99]. Across all the 5 waves, children from wealthier households were less likely to experience child hunger than children from the poorest households. The intensity of the likelihood would decrease as household wealth increases (Table 4). For instance in wave 1; Poorer [0.54(95% CI: 0.53–0.54)], Middle [0.53(95% CI: 0.53 0.53)], Richer 0.52(95% CI: 0.52–0.52)] and Richest [0.21(95% CI: 0.21–0.21)] it can de deduced the odds ratios are decreasing as household wealth increases.

Children who stayed in traditional/mud waves 1 [1.05(95% CI:1.04–1.05)], wave 3 [1.95(95% CI: 1.93–1.96)], wave 4 [1.13(95% CI: 1.13–1.14)] and wave 5 [1.66(95% CI: 1.65–1.66)] and informal shack type of dwelling were more likely to experience the child hunger for wave 1 [1.77(95% CI: 1.76 1.77)], wave 3 [3.37(95% CI: 3.34 3.40)], wave 4 [2.17(95% CI: 2.16–2.18)] and 5 [2.66(95% CI: 2.65 -2.68] except in wave 2 in which children who dwelled in informal shacks [0.61(95% CI: 0.60–0.61)] were less likely to experience the child hunger compared to children who dwelled in houses/flats. The regression results also showed that as household size increased, so was the likelihood of the children experiencing a child hunger across the 5 waves.

### Concentration indices

All concentration indices for child hunger for children across households for waves 1–5 were negative, meaning that children from poor households were hungry. Also, the intensity of the pro-poor indices increased for the period under review [wave 1 (-0.149), wave 2 (-0.102), wave 3 (-0.153), wave 4 (-0.182) and wave 5 (-0.211)] (Table 5). All the Erreygers Normalised concentration indices were statistically significant at 95% confidence interval.

**Table 5:** Erreygers Normalised Concentration Indices (ENCI) for the adult reported child hunger in South Africa for Wave 1–5

	***Wave 1***	***Wave 2***	***Wave 3***	***Wave 4***	***Wave 5***
Index value	-0.149	-0.102	-0.153	-0.182	-0.211
Std. error	0.015	0.014	0.027	0.017	0.019
*p*-value	0.000	0.000	0.000	0.000	0.000

**Fig. 1:**
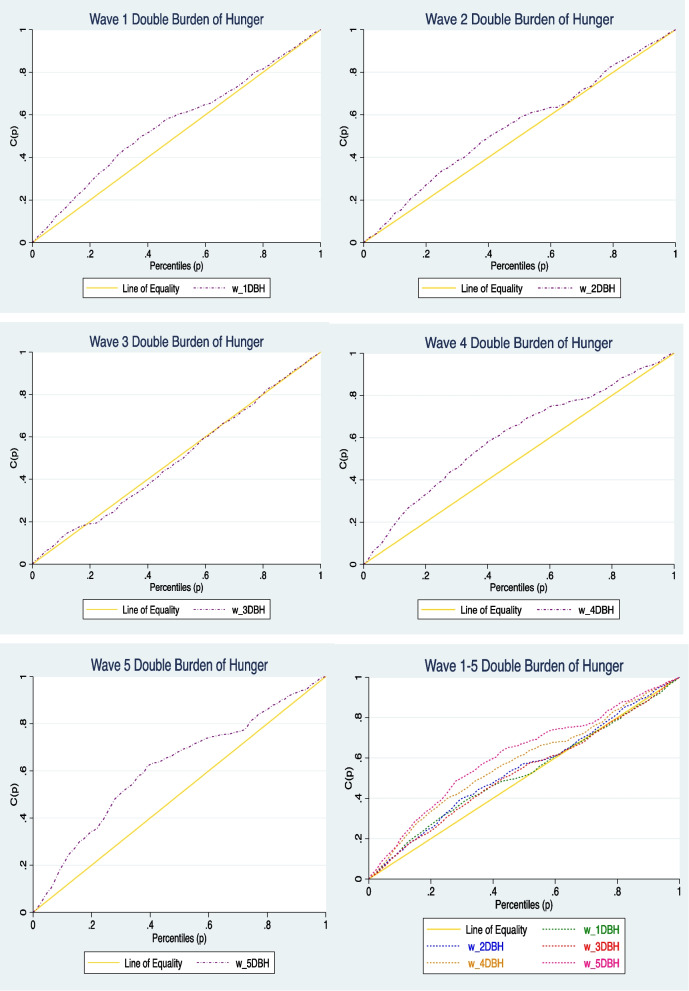
**a-e** show the concentration curves for adult reported child hunger per each wave, and f shows the concentration curves for adult reported child hunger for all the waves on one graph

Figure 1; (a, b, c, d & e) show concentration curves for adult reported child hunger relative to household income for the 5 waves. The computed concentration curves concur with the indices that the socioeconomic inequalities were pro-poor. We only computed the dominance test for wave 3 concentration curves as it crossed the 45° line (line of equality) at some points. The dominance test gives a clearer picture of the cumulative population distribution along the concentration curves [46]. Furthermore, the dominance test computed was non-dominant, meaning that the concentration curves dominated the 45° line. Therefore, the dominance test results mean that the concentration curve (Fig. 1; c) concurs with the concentration indices findings (Table 5). Figure 1; f shows all the concentration curves plotted in one graph, and all showed pro-poor inequalities relative to the adult reported child hunger.

### Decomposition analysis

To better understand what was driving the socioeconomic inequalities, the study decomposed the Erreygers Normalised Concentration Indices (ENCI). Across all the 5 waves the results showed that race [wave 1(21.11%); wave 2(-40.34%); wave 3(60.74%); wave 4(-26.62%) & wave 5(19.14%)], socioeconomic status [wave 1(91.55%); wave 2(127.78%); wave 3(56.56%); wave 4(120.75%) & wave 5(109.03%)] and dwelling type [wave 1(6.77%); wave 2(-5.22%); wave 3(12.49%); wave 4(7.81%) & wave 5(7.62%)] were significant drivers of the adult reported child hunger in South Africa (Table 6). Also to note is that race and socioeconomic inequalities were driving pro-rich inequalities while dwelling type was driving pro-poor inequalities relative to the adult reported child hunger.

**Table 6:** Decomposition analysis of the drivers of socioeconomic inequalities relative to adult reported child hunger in South Africa Wave (1–5)

	**Wave 1**			**Wave 2**			**Wave 3**		
	**Elasticity**	**Concentration Index**	**Contribution (%)**	**Elasticity**	**Concentration Index**	**Contribution (%)**	**Elasticity**	**Concentration Index**	**Contribution (%)**
Employment status	0,008	0,090	-2,055	0,002	0,094	-0,890	0,000	0,000	0,000
Residence status	-0,003	0,017	0,117	0,030	0,051	-6,354	0,020	0,041	-2,019
Race	-0,055	0,141	21,113	-0,081	0,120	-40,339	-0,193	0,125	60,739
Socioeconomic Status (SES)	-0,084	0,400	91,545	-0,077	0,401	127,775	-0,069	0,328	56,563
Dwelling Type	0,010	-0,239	6,771	-0,006	-0,222	-5,217	0,018	-0,275	12,485
Electricity access	-0,035	-0,012	-1,109	0,062	-0,010	2,669	-0,074	-0,012	-2,233
Piped-water access	0,013	-0,036	1,236	0,028	-0,034	3,913	0,022	-0,041	2,222
Respondent's Education Level	-0,034	0,028	-2,555	-0,074	0,031	9,594	-0,031	0,026	-2,081
Gender	0,037	-0,015	-1,552	0,009	-0,023	0,810	0,057	-0,051	-7,242
Household size	0,037	-0,025	-2,510	0,019	-0,017	1,374	0,016	-0,073	2,890
Respondent’s age	0,035	0,023	-2,180	0,028	0,024	-2,730	0,054	0,016	-2,205
***Residuals***	***-8,423***			***9,396***			***5,852***		

	**Wave 4**			**Wave 5**		
	**Elasticity**	**Concentration Index**	**Contribution (%)**	**Elasticity**	**Concentration Index**	**Contribution (%)**
Employment status	-0,001	0,110	0,271	-0,013	0,117	2,999
Residence status	-0,013	0,041	1,227	0,033	0,042	-2,742
Race	-0,096	0,118	-26,624	-0,073	0,134	19,136
Socioeconomic Status (SES)	-0,109	0,390	120,751	-0,144	0,387	109,031
Dwelling Type	0,015	-0,222	7,805	0,019	-0,208	7,621
Electricity access	-0,059	-0,015	-2,087	-0,078	-0,014	-2,152
Piped-water access	0,000	-0,038	-0,027	-0,018	-0,040	-1,427
Respondent's Education Level	-0,041	0,025	2,339	0,008	0,030	-0,449
Gender	0,021	-0,032	1,568	0,051	-0,038	3,824
Household size	0,061	-0,010	1,468	0,072	0,000	-0,048
Respondent’s age	0,010	0,013	-0,292	-0,070	0,009	1,261
***Residuals***	***-6,400***			***1.216***		

## Discussion

This paper aimed to examined whether adult reported child hunger and socioeconomic inequalities in South Africa were exacerbated during the COVID-19 pandemic due to various lockdown regulations that affected the economic status of the population. Nonetheless, socioeconomic inequalities in adult reported child hunger remain an important measure for determining chronic and hidden hunger among children [47]. Undoubtedly, the COVID-19 pandemic resulted in a sharp rise in unemployment and unprecedented increases in poverty, food insecurity, and declining health conditions around the world [48]. Families of young children, adolescents, pregnant and lactating women need to be protected from the ongoing long-term pandemic and the aftershocks that are likely to continue for years to come. The COVID-19 pandemic has demonstrated the unpreparedness of people to protect them from hunger, food insecurity, malnutrition and health insecurity in the looming global crisis. In recent decades, South Africa has adopted several food and nutrition guidelines to improve food security for children [49]. Although some children's health indicators did not change significantly, children's self-reported hunger was reported to decrease [5].

Poverty, unemployment and hunger have reportedly increased sharply in the wake of COVID-19-related lockdowns. With 47% of households running out of money to buy food in May/ June 2020 (wave 1), while child and adult hunger increased to 15% and 22% [27]. The proportion of households running out of money to buy food declined to 37% by July/August 2020 (wave 2) due to the introduction of the caregiver and COVID-19 relief grants [5]. However, hunger levels remained significantly higher than pre-COVID-19 levels [5]. Our study reported similar results as the prevalence of hunger varied in five waves. However, adult reported child hunger was highest in the first wave and lowest in the second wave.

Like the rest of the world, to stem the spread of the COVID-19 pandemic, South Africa imposed restrictions on social mobility and interaction, enacting nationwide lockdowns. The gradual, risk-adjusted reopening of the South African economy following formal lockdowns invariably resulted in significant welfare losses to individuals and households [50]. The latter pandemic-related shocks to employment and working hours resulted in low-wage earnings which then exacerbated already high levels of poverty and inequality in South Africa. Without question the aforementioned negative shocks to the economy by the covid-19 pandemic, caused an immediate drop in economic activities, which was then followed by medium to long-term economic consequences [51]. The burden of these shocks was not shared equally by society. To put the latter into perspective, high-income professionals and administrators were able to maintain physical distancing, but in densely populated areas where work is concentrated, mining, manufacturing, retail, and service workers were affected [52]. The reviewed literature suggested that traditionally more vulnerable groups, such as women, black Africans, youth and uneducated groups, were disproportionately affected [53].

Taking into account all the indirect effects of the covid-19 lockdowns in South Africa, gross domestic product (GDP), which is rightly regarded as flows of goods and services, fell by about a third, with indirect effects accounting for most of the decline [54]. Employment also plummeted as a result of the covid-19 pandemic in South Africa. Low-skilled and uneducated workers were the most affected, with the net effect of the covid-19 shocks most severe for poorer and more vulnerable households [50–54]. These negative economic shocks alone were large enough to make many households food insecure and hungry. This reduced ability to afford food was caused by severe household income shocks rather than drought-like food availability shocks [54].

Therefore, lockdown levels in South Africa had a huge impact on hunger, with a strict lockdown (Level 5) being introduced after three days of warning. At that time, most people were not paid wages or salaries, and many households were unable to afford food [55]. Food was only available in supermarkets as all informal activities, including the sale of food, had ceased. As a result, groceries were only accessible to those with transportation or within walking distance of a supermarket. However, most South Africans living in townships and rural areas were unable to access food due to the proximity of farms and supermarkets [55]. Most urban township residents usually buy their groceries from local street vendors and small 'spaza' shops [56]. During the severe lockdown, they were unable to travel to the city center because public transport was not available. It was exacerbated by the loss of income caused by those in the formal economy [55]. This could explain the higher rate of adult reported child hunger seen in urban children compared to rural children.

There is ample evidence in the literature that social factors such as education, employment status, income level, gender, and ethnicity have significant effects [57–59]. All countries, whether low-middle-income or high-income, have large disparities in health status among different social groups [60]. It has been emphasized that economic growth is necessary, but not sufficient, to sustain progress in reducing poverty and hunger [57]. It has been reported that about three-quarters of the world's poor live in rural areas, making up a large proportion of the hungry and malnourished in developing countries [58]. The lower the socioeconomic status, the higher the health risk. Our research also found that children's chances of suffering from hunger decreased as household wealth increased.

The severity of parent-poor inequality in adult reported child hunger in South Africa can be explained by the sharp rise in food prices before, during and after lockdown, with household staple food baskets increasing by about 14.4%. [November 2019, (R3,106,42)—November 2020, (R3,554,64)] [14]. This also reflects an important argument repeatedly emphasized in the literature that socioeconomic status is a major determinant of health inequalities [48, 59, 61–65]. A sharp increase in household food baskets was associated with a sharp rise in hunger levels in South Africa.

School feeding programs have long been an important safety net for starving children in resource-poor settings [66]. Implementation of school health programs has led to improved public health, particularly among vulnerable groups such as the poorest children, girls and children in conflict, through improved nutrition and learning. Unfortunately, most schools around the world have closed during his COVID-19 pandemic, leaving children more vulnerable and hungry [66]. The growing poverty-related hunger inequality among children in South Africa can therefore be attributed to the safety nets disrupted by the COVID-19 pandemic due to strict lockdown measures.

### Policy recommendations

Health inequalities are differences in health status or distribution of health resources between different population groups that result from the social conditions in which people are born, grow, live, work and age [65]. Health inequalities are unfair and can be reduced with the right mix of public policies. COVID-19 has had a negative impact on the health and well-being of children around the world, especially in low and middle income countries (LMICs). The pandemic has pushed families into food insecurity and hunger [9]. This study shows that COVID-19 has magnified existing hunger inequalities among children in South Africa. We therefore suggest investing in research on the impact of pandemics on food and nutrition in developing countries and the effective implementation of equitable social protection programs and policies.

Recognize that hunger stems from inequalities across sectors. Therefore, there is need to develop an equitable, effective and rapid response system to prevent or reduce hunger. This can be done by building a framework of a complex adaptive systems for South Africa and the international community. However, when developing an equitable emergency system, special attention should be paid to households with children, young people and pregnant and lactating women.

The widespread impact of the COVID-19 pandemic is adversely affecting hunger, food and nutrition security, the health and well-being of families with young children, and pregnant and lactating women. It will probably last for years [66, 67]. It is therefore time to dig deeper to understand the root causes of inequality and implement coordinated policies to ensure all pre-COVID-19 public health advances are not undermined. Adult reported child hunger is a subjective metric, thus other important aspects of food security, such as dietary diversity and consumption of nutritious foods, are not captured [26]. Dietary diversity and consumption of nutrient-rich foods are important indicators for assessing healthy growth in children, especially in early childhood. However, they still lack access to adequate nutritious food and are at risk of malnutrition. Child hunger should therefore be assessed holistically, not just subjectively.

In addition to economic challenges, South Africa's lockdown restrictions have indirectly impacted access to essential goods and services in a number of ways [8]. Most of the food was wasted as the food system was disrupted, making it difficult to get food to markets and reducing food demand.

## Conclusion

This study describes adult reported child hunger and associated socioeconomic inequalities during the Covid-19 pandemic. The increasing prevalence of child hunger, especially among urban children, and the observed poverty inequality necessitate multisectoral pandemic shock interventions now and in the future, especially for urban households. Developing countries' budgets have already been depleted, and the price of inaction to alleviate child hunger will be long overdue.

## Data Availability

All the data is freely and publicly available on https://www.datafirst.uct.ac.za/dataportal/index.php/catalog/NIDS-CRAM, allowing others to replicate the results we report here. Access to the data can be granted through the NIDS-CRAM data repository.

## Footnotes

1 NIDS ran between 2008 and 2017 with a nationally representative sample of over 28,000 individuals in 7,300 households over time and reported on their livelihoods and wellbeing across South Africa. The latest round of in-person data collection (Wave 5) was conducted in 2017 (SALDRU, 2018). The follow-up telephonic survey (NIDS-CRAM) was available in 10 official languages.
